# Human umbilical vein endothelial cells-derived exosomes enhance cardiac function after acute myocardial infarction by activating the PI3K/AKT signaling pathway

**DOI:** 10.1080/21655979.2022.2056317

**Published:** 2022-03-31

**Authors:** Wei Liu, Yu Feng, Xuehua Wang, Jiaxing Ding, Huili Li, Hongquan Guan, Zhijian Chen

**Affiliations:** Department of Cardiology, Union Hospital, Tongji Medical College, Huazhong University of Science and Technology, Wuhan, Hubei, China

**Keywords:** Human umbilical vein endothelial cells, exosomes, acute myocardial infarction, cardiac function, apoptosis, PI3K/AKT signaling pathway

## Abstract

Currently, acute myocardial infarction (AMI) is one of the leading causes of human health issues worldwide. The sudden and continuous occlusion of the coronary artery results in myocardial hypoxic-ischemic necrosis, which is accompanied by inflammatory infiltration and fibrosis, leading to pathological cardiac remodeling. Exosome-based therapy is a promising cell-free approach for repairing the ischemic myocardium. This study aimed to explore the effects and mechanism of human umbilical vein endothelial cells (HUVECs)-derived exosomes on AMI. The results indicated that the localized injection of HUVECs-derived exosomes in the infarcted area could significantly improve cardiac function in AMI mouse models. It could also ameliorate myocardial fibrosis and decrease infarct size after AMI. Additionally, HUVECs-derived exosomes had cardioprotective effects on the H9C2 cells in hypoxic culture conditions, including increased cell viability and decreased lactate dehydrogenase (LDH) release. In both the *in-vivo* and *in-vitro* experiments, HUVECs-derived exosomes could effectively inhibit cardiomyocyte apoptosis. The low expression levels of Bcl-2–associated X protein (Bax) and cleaved caspase-3, high expression levels of B-cell lymphoma 2 (Bcl-2), phosphorylated phosphatidylinositol 3-kinase (p-PI3K), and phosphorylated protein kinase B (p-AKT) were detected in AMI mouse models treated with HUVECs-derived exosomes *in-vivo*. In conclusion, HUVECs-derived exosomes effectively enhanced cardiac function after AMI and inhibited cardiomyocyte apoptosis, which might be regulated through the phosphatidylinositol 3-kinase (PI3K)/ protein kinase B (AKT) signaling pathway.

## Graphical Abstract

**Figure:**
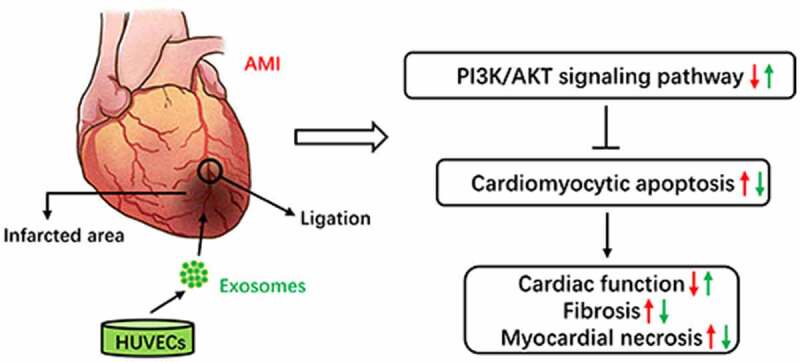

## Introduction

Ischemic heart disease (IHD) is one of the major threats to public health worldwide. The latest report on the global burden of cardiovascular disease shows 197 million prevalent cases and 9.14 million death cases of IHD [1]. Acute myocardial infarction (AMI), as a severe type of ischemic heart disease, is the leading cause of death and disability. The blood supply to the coronary artery is sharply reduced or halted after the temporary or persistent occlusion of a coronary artery. The myocardium’s corresponding areas experience sustained and severe ischemia, causing irreversible acute necrosis [2]. After AMI, sometimes cardiac remodeling can occur, which is linked to a series of complications, such as ventricular aneurysm, malignant arrhythmia, and heart failure [3]. Therefore, a potential and effective method for treating AMI and left ventricular dysfunction is needed.

The cardiomyocyte apoptosis occurs throughout the acute, subacute, and chronic phases after AMI [4]. Several studies show that reducing cardiomyocyte apoptosis can improve AMI-induced cardiac remodeling and dysfunction [5–7]. Consequently, the inhibition of cardiomyocyte apoptosis has been regarded as a potential therapeutic target for the treatment of AMI patients. Previous research has revealed that the phosphatidylinositol 3-kinase (PI3K)/protein kinase B (AKT) signaling pathway plays a significant role in apoptosis [8]. When PI3K is activated by extracellular signals, such as growth factors, cytokines, and hormones, it will be phosphorylated and act on a downstream effector molecule called protein kinase B (AKT). Then the phosphorylation sites of AKT are exposed, leading to the activation of the signal transduction pathway [8]. The activated AKT increases the expression of Bcl-2 (B-cell lymphoma 2) by phosphorylating the Bcl-2 family members to protect the cells from apoptosis [9].

Exosomes are double-layered lipid vesicles with a diameter of 30–150 nm released by cells [10]. They carry a variety of cargo molecules, including proteins, lipids, metabolites, DNA, and RNA [11]. Moreover, exosomes are abundantly present in various body fluids, including blood, urine, amniotic fluid, and breast milk [12]. As the local and remote mediators of intercellular signal transduction, exosomes are involved in numerous physiological and pathological processes of the human body and related to the occurrence and development of cardiovascular diseases [13]. Previous studies have shown that exosomes participate in the repairing process after AMI by preventing cardiomyocyte apoptosis, promoting angiogenesis, inhibiting inflammatory response, and mobilizing bone marrow stem cells [14]. Hence, exosomes have an enormous potential for the treatment of AMI.

It has been reported that the localized injection of human umbilical vein endothelial cells (HUVECs) into the infarction and border zone of AMI mouse models can improve the cardiac remodeling by deactivating the matrix metalloproteinases (MMPs) and increasing the expression of tissue inhibitor of metalloprotease 1 and 3 (TIMP1 and TIMP3) [15]. Moreover, the localized injection of HUVECs contributes to macrophage infiltration and angiogenesis in the infarcted area, enhancing cardiac function and increasing coronary blood flow [16]. These cardioprotective effects may be related to the paracrine signaling of HUVECs.

As mentioned above, transplanting HUVECs into the infarcted area can ameliorate cardiac remodeling and restore cardiac function in AMI animal models. However, HUVECs-derived exosomes’ involvement in AMI has not been demonstrated yet. This research aimed to explore the effects and mechanism of HUVECs-derived exosomes on AMI in mice. We hypothesized that HUVECs-derived exosomes probably played a therapeutic role in AMI, and this work might provide a novel perspective for the treatment of AMI.

## Materials and methods

### Cell culture

HUVECs and rat H9C2 cell lines were purchased from Procell Life Science and Technology Co., Ltd. (Wuhan, China). The cell cultures were maintained in a high-glucose Dulbecco’s Modified Eagle Medium (DMEM, Gibco, Grand Island, NY, USA), containing 10% fetal bovine serum (FBS, Gibco, Grand Island, NY, USA) and 1% penicillin/streptomycin (Gibco, Grand Island, NY, USA). All the cells were placed in a humid incubator at 37°C with a 5% CO_2_ concentration. The culture medium was replaced every two days. When the cells grew to 80% to 90% cell confluence, 0.25% trypsin-ethylenediaminetetraacetic acid (trypsin-EDTA, Gibco, Grand Island, NY, USA) was used for cell passaging. HUVECs and H9C2 cells from the third to sixth passage were selected for this study. The cells after the twelfth passage were discarded. The cells from the same passage were resuscitated at intervals to ensure stability.

### Exosomes isolation

Exosomes were isolated by ultracentrifugation [17]. HUVECs from the third to sixth passage were cultured in a high-glucose DMEM supplemented with 10% FBS and 1% penicillin/streptomycin. The culture medium was replaced when the HUVECs reached approximately 80% cell confluency. The cells were washed with sterile phosphate-buffered saline (PBS) three times and cultured in a high-glucose DMEM, containing 10% exosome-free FBS and 1% penicillin-streptomycin for 48 h. Then, the culture medium was collected for exosomes isolation. All the centrifugations were performed at 4°C. First, the culture medium was centrifuged at 300 g for 10 min and 3,000 g for 15 min to remove the dead cells and cellular debris. Then, the supernatant was centrifuged at 12,000 g for 30 min and filtered through a 0.22-μm microfilter for removing the large micro-vesicles. For precipitating the exosomes, the supernatant was poured into 26.3-mL polycarbonate bottles and centrifuged at 100,000 g for 120 min using an Optima XPN-100 Ultracentrifuge (Beckman counter, lnc, California, USA) equipped with a Type 70 Ti rotor. The supernatant was discarded, and the exosomes pellet was collected. Finally, the exosomes pellets were resuspended in sterile PBS. Bicinchoninic acid (BCA) protein assay (Thermo Fisher Scientific, Waltham, USA) measured exosomes’ protein concentration. The isolated exosomes were stored in liquid nitrogen for the subsequent research.

### Transmission electron microscopy (TEM)

The isolated exosomes were fixed with 2.5% glutaraldehyde at 4°C overnight. Then, a 20-μL exosomes suspension was loaded on a carbon-coated grid with a pore diameter of 2 nm for 2 min and then negatively stained with 2% uranyl acetate solution. The excess staining solution on the grid was washed off with PBS. The remaining liquid on the grid was air-dried, and the images of exosomes’ morphology were obtained using a Tecnai G2 20 TEM (FEI, Eindhoven, Netherlands).

### Nanoparticle tracking analysis

The particle size and concentration of exosomes were characterized using a NanoSight NS300 system (Malvern Panalytical, Malvern, UK) equipped with a 405-nm laser beam. The isolated exosomes were diluted with sterile PBS to reach the recommended measurement range (10^6^ to 10^9^ particles/mL). The diluted exosomes solution was injected slowly into the instrument channel using a syringe. Exosomes were illuminated with a laser beam, resulting in a dynamic light scattering. The light scattered by exosomes was captured using a camera, which indicated the paths of exosomes under Brownian motion. The movement of exosomes was recorded on a 30-sec video, which was analyzed using the NTA software (NanoSight, version 3.0). The particle size and the concentration of exosomes were calculated using the Stokes-Einstein equation [18].

### Animal models

Eight-week-old male C57BL/6 mice, weighing 22–24 g, were purchased from Beijing Vital River Laboratory Animal Technology Co. Ltd (Beijing, China). All the mice were raised in a specific pathogen-free (SPF) animal house of Tongji Medical School Laboratory Animal Center. The mice were kept at 25°C with 55% humidity and 12/12 h of light/dark cycle. The mice were allowed to eat and drink *ad libitum*.

### Ethics statement

All the animal studies were performed following the Guide for the Care and Use of Laboratory Animals issued by the National Institutes of Health (NIH publication No. 86–23, revised 1985) and approved by the Institutional Animal Care and Use Committee at Tongji Medical College, Huazhong University of Science and Technology (No.2647).

### AMI mouse models and exosomes injection

The C57BL/6 mice (male, eight weeks, 22–24 g) were randomly divided into three groups: sham group (n = 6), receiving sham operation; AMI + PBS group (n = 6), receiving AMI induction with the localized injection of PBS; and AMI + exosomes (Exo) group (n = 6), receiving AMI induction with the localized injection of HUVECs-derived exosomes. The AMI mouse models were established as described previously [19]. The mice anesthetized with pentobarbital sodium (50 mg/kg) were orally intubated and mechanically ventilated (2.6 ml tidal volume, 110/min respiration rate, and 1:3 ventilatory ratio). Then, their hearts were fully exposed by performing the thoracotomy between the third and fourth intercostal space, and the left anterior descending (LAD) coronary artery was permanently ligated using a 6–0 suture. Finally, their chests were closed using a 4–0 suture. The sham group mice underwent the same surgical procedure except for the permanent LAD coronary artery ligation. Then, 25 μL of HUVECs-derived exosomes (2 μg/μL) or sterile PBS were injected into the infarcted areas. The mice were sacrificed 7 days or 28 days after AMI, and their hearts were harvested for later analyses. To confirm the successful induction of AMI in mice, BL-420 F biological signal acquisition and processing system (Taimeng, Chengdu, China) was used to record the electrocardiograms of mice before and after LAD coronary artery ligation. One day after surgery, the two most significant indicators of cardiac function in mice, including ejection fraction (EF) and fractional shortening (FS), were assessed. Some mice were sacrificed using anesthetic overdose, and their hearts were separated from their thoraxes. Then, the heart specimens were equally cut into four sections along their long axis. These sections were placed into 2% 2,3,5-triphenyl tetrazolium chloride (TTC) solution (Solarbio, Beijing, China) at 37°C for 15 min in the dark. Then, the sections were assessed macroscopically and photographed.

### Hematoxylin and eosin (HE) staining

The cardiac tissues were fixed with 4% paraformaldehyde and dehydrated with gradient alcohol. After dehydration, the specimens were embedded in paraffin and sectioned into 5-6-μm thick slices. Finally, these sections were stained with HE staining solution following the standard procedures. The pathological changes in the cardiac tissues were observed under a light microscope (Olympus Corporation, Tokyo, Japan).

### Masson’s trichrome staining

Briefly, the cardiac tissues were fixed, embedded, and cut into slices similar to those performed for HE staining. Then, these slices were stained with Masson’s trichrome following the standard procedures. Images were captured for analysis under a light microscope. ImageJ software was used to evaluate the extent of fibrosis. The percentage of fibrosis area in the left ventricular was calculated as the ratio of fibrosis area to the whole left ventricular region [20]. The epicardial and endocardial scar circumference were traced manually and measured by computerized planimetry [21].

### Echocardiographic assessment

The cardiac function of mice was evaluated by transthoracic echocardiography [22] (Vevo 3100 imaging system, Vevo 3100, FUJIFILM VisualSonics lnc., Toronto, ON, Canada). Two experienced echo-cardiologists performed the M-mode echocardiography. At the papillary level, the echocardiography parameters, including left ventricular ejection fraction (LVEF), left ventricular fractional shortening (LVFS), left ventricular end-systolic diameter (LVESD), and left ventricular end-diastolic diameter (LVEDD), were recorded from the mice anesthetized with isoflurane. All the data were measured in five consecutive cardiac cycles.

### Co‑culture experiment

H9C2 cells were seeded in 24-well plates (1 × 10^5^ cells/well) or 96-well plates (5 × 10^3^ cells/well) and divided into three groups, including the Normoxia group (n = 6), Hypoxia + PBS group (n = 6), and Hypoxia + Exo group (n = 6). The cells in the Normoxia group were cultured under normal conditions. At the same time, the cells in the other two groups were cultured in low-glucose and serum-free DMEM (Gibco, Grand Island, NY, USA) without penicillin/streptomycin. Then, the Hypoxia + PBS group and Hypoxia + Exo group cells were incubated with PBS and HUVECs-derived exosomes, respectively, for 24 h and placed in a hypoxic environment at 37°C with 94% N_2_, 1% O_2_, and 5% CO_2_.

### Flow cytometry analysis of apoptotic cells

The apoptosis of H9C2 cells was assessed using a FITC Annexin V Apoptosis Detection Kit I (BD Biosciences, New Jersey, USA). The cells were collected by washing with 0.25% trypsin, followed by washing with ice-cold PBS three times. Then, the cells were incubated with FITC Annexin V and propidium iodide (PI) for 15 min at room temperature in the dark. The cells’ apoptotic rate was detected using a flow cytometer (BD Biosciences, New Jersey, USA). The data was analyzed using the FlowJo software version 10. The apoptotic index was calculated as the percentage of the early apoptotic cells (only Annexin V positive cells) and the late apoptotic cells (Annexin V and PI dual positive cells) [23].

### Cell viability

Cell viability was determined using a Cell Counting Kit-8 (Biosharp, Hefei, China). After completing the co-culture experiment, 10 μL of Cell Counting Kit-8 (CCK-8) solution was added to each well containing H9C2 cells and incubated for 2 h at 37°C. The absorbance of formazan was measured at a 450-nm wavelength using a microplate reader (PerkinElmer, Waltham, USA).

### Lactate dehydrogenase (LDH) activity

LDH activity was measured using an LDH Cytotoxicity Assay Kit (Beyotime, Shanghai, China). After completing the co-culture experiment, the supernatant of H9C2 cell culture was transferred to a new microplate (120 μL/well). Then, a 60 μL LDH detection working solution was added to each well, following the manufacturer’s instructions. The absorbance of formazan was measured at a 490-nm wavelength using a microplate reader after incubation for 30 min at 37°C in the dark.

### Labeled exosomes

To evaluate whether the exosomes were internalized by rat H9C2 cells, the isolated exosomes were labeled using a PKH26 Red Fluorescent Cell Linker Kit (Sigma-Aldrich Chemical Company, St Louis, MO, USA) and isolated again through ultracentrifugation. Then, the labeled exosomes were incubated with H9C2 cells for 6 h, 12 h, and 24 h. After incubation, the cells were washed with sterile PBS and stained with 4’, 6-diamidino-2-phenylindole (DAPI, Beyotime, Shanghai, China) for 5 min at room temperature. Images were captured under a fluorescence microscope (Olympus Corporation, Tokyo, Japan).

### TUNEL staining analysis for apoptosis

The cardiomyocyte apoptosis in cardiac tissue was determined using a terminal deoxynucleotidyl transferase (TdT)-mediated dUTP nick-end labeling (TUNEL) Apoptosis Detection Kit (Beyotime, Shanghai, China). The cardiac tissues were made into paraffin sections described in HE and Masson’s trichrome staining. Then, these sections were stained using the TUNEL assay kit following the manufacturer’s instructions. The apoptotic cell nuclei were TUNEL-positive (green), and all the cell nuclei were labeled with DAPI (blue). The slides were observed under a fluorescence microscope. Five randomly selected microscopic fields (200X) for each slide were selected to count the TUNEL-positive and DAPI-stained cells. The percentage of apoptotic cells was calculated as the ratio of TUNEL-positive cells to the whole DAPI-stained cells.

### Western blotting

The total protein contents were extracted from the HUVECs-derived exosomes, HUVECs, and cardiac tissues. Western blotting was performed following the standard procedures [24]. The following primary antibodies were used: Calnexin (1:2000, Proteintech, Rosemont, USA), tumor susceptibility gene 101 (TSG101, 1:500, Proteintech), CD63 (1:1000, Abcam, Cambridgeshire, UK), CD81 (1:1000, Abcam), B Cell Lymphoma-2-associated X protein (Bax, 1:5000, Proteintech), Bcl-2 (1:1000, Proteintech), cleaved caspase-3 (1:1000, Abcam), PI3K (1:5000, Proteintech), phosphorylated phosphatidylinositol 3-kinase (p-PI3K, 1:500, Abcam), AKT (1:5000, Proteintech), phosphorylated protein kinase B (p-AKT, 1:2000, Proteintech), and β-actin (1:5000, Proteintech). The following secondary antibodies were used: Goat anti-rabbit antibody (1:3000, Ant Gene, Wuhan, China) and Goat anti-mouse antibody (1:3000, Ant Gene).

### Statistical analysis

All the data were described as mean ± SD and analyzed using Statistical Product and Service Solution (SPSS) software version 22.0 (IBM Corporation, Armonk, NY). The statistical significance of differences among different groups was assessed using one-way analysis of variance (ANOVA) followed by LSD post hoc least significant difference test. *P*-values <0.05 were considered statistically significant.

## Results

In this study, HUVECs-derived exosomes were hypothesized to be therapeutic in AMI. The effects and mechanism of HUVECs-derived exosomes on treating AMI in mice were further investigated. For this purpose, we established AMI mouse models *in vivo* and hypoxic H9C2 cell models *in vitro*. HUVECs-derived exosomes were administered to them, respectively. Cardiac function, myocardial pathological changes, cardiomyocyte apoptosis, and the expression of Bax, Bcl-2, cleaved caspase-3, PI3K, p-PI3K, AKT, and p-AKT were evaluated.

### Separation process and identification of exosomes derived from HUVECs

**Figure 1.**
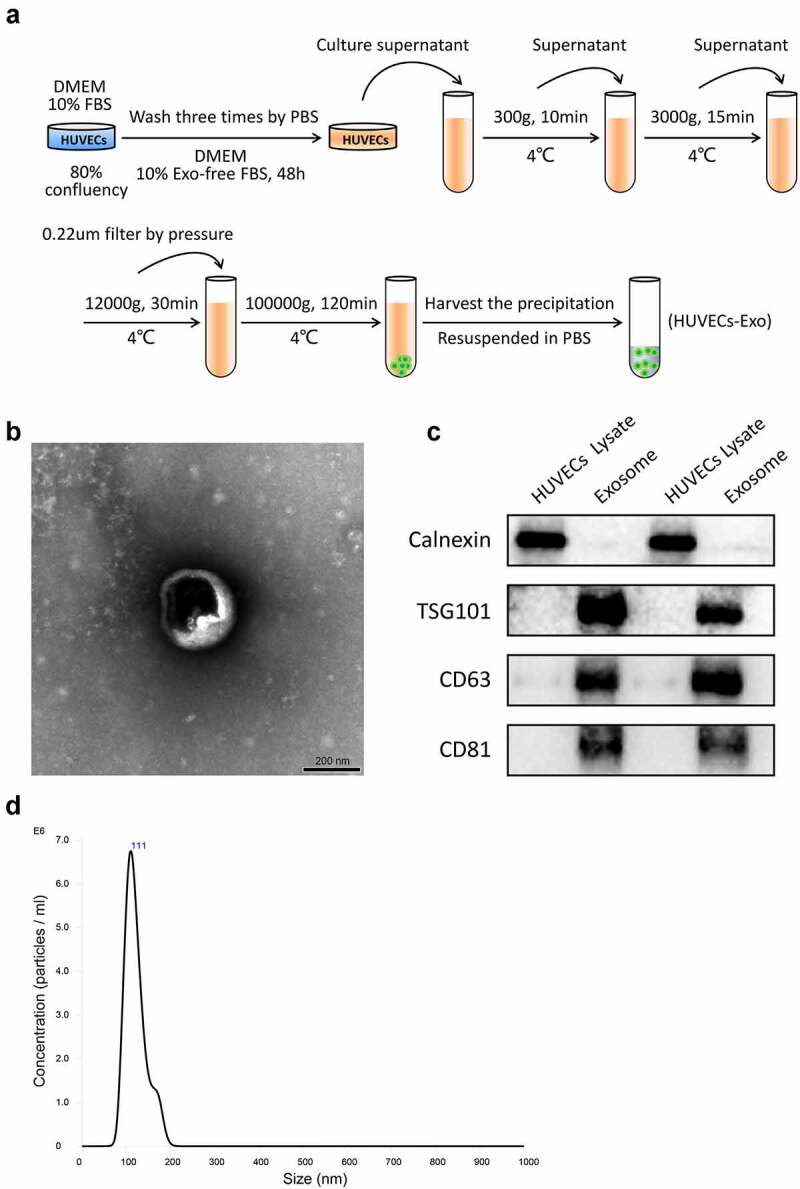
Separation process and Identification of exosomes derived from HUVECs. (a) Isolation protocol of exosomes from HUVECs. (b) Morphology of exosomes observed under a transmission electron microscope (scale bar = 200 nm). (c) Expression of Calnexin, TSG101, CD63, and CD81 in the HUVECs lysate or exosomes detected using Western blotting. (d) The particle size and concentration of exosomes were characterized using nanoparticle tracking analysis. Abbreviations: HUVECs, human umbilical vein endothelial cells; DMEM, Dulbecco’s modified eagle medium; FBS, fetal bovine serum; PBS, phosphate-buffered saline; and Exo, exosome.

To obtain HUVECs-derived exosomes for subsequent experiments, HUVECs were cultured in a high-glucose DMEM, containing 10% exosome-free FBS and 1% penicillin-streptomycin for 48 h. Then, exosomes were isolated from the culture medium of HUVECs using ultracentrifugation (Figure 1a). The observation of HUVECs-derived exosomes under TEM showed that exosomes were round or oval membranous vesicles resembling a saucer or cup (Figure 1b). The presence of exosomes specific marker proteins (TSG101, CD63, and CD81) was verified by western blotting. In contrast, the expression of intracellular protein Calnexin was almost undetectable compared to that of the HUVECs (Figure 1c). The NTA software showed that the diameter of HUVECs-derived exosomes ranged from 100–150 nm with the mean particle concentration of 3.39 × 10^8^ particle/mL (Figure 1d).

### Establishment of an AMI mouse model

**Figure 2.**
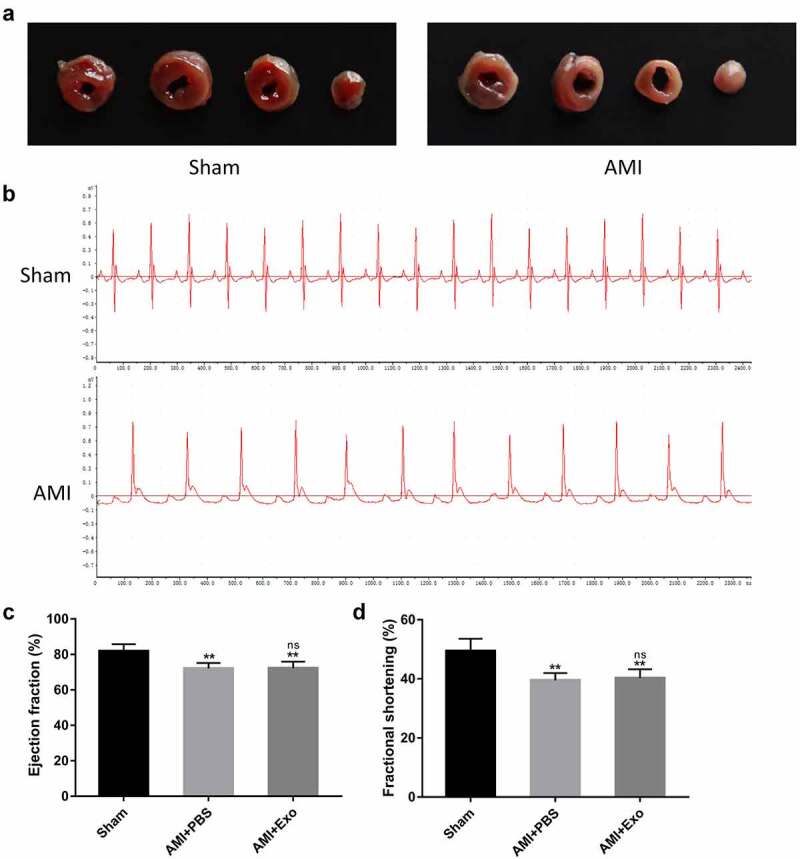
Establishment of a mouse AMI model. (a) Representative images of heart sections one day after LAD coronary artery ligation using TTC staining. (b) Electrocardiograms of mice were recorded using the BL-420 F biological signal acquisition and processing system. (c-d) One day after LAD coronary artery ligation, cardiac function-related parameters of the mice were assessed using transthoracic echocardiography (n = 6 per group). Statistical data were described as mean ± SD, ANOVA was used followed by LSD post hoc least significant difference test; **P < 0.01 vs. Sham group; ns meant no significance vs. AMI + PBS group. Abbreviations: AMI, acute myocardial infarction; PBS, phosphate-buffered saline; Exo, exosome; and ns, no significance.

The AMI mouse models were established *in-vivo* by ligating the LAD coronary artery. TTC staining and electrocardiograms were used to confirm the successful establishment of AMI mouse models. TTC staining revealed that the infarcted area of the left ventricle was pale, while the non-infarcted area of that was red (Figure 2a). The electrocardiogram signals recorded by the biological signal acquisition and processing system suggested that the ST-segment elevation and pathological Q waves were detected as early as 24 h (Figure 2b). After one day of surgery, the analysis of cardiac function-related indicators showed that the LVEF and LVFS did not differ between the AMI + PBS and AMI + Exo groups, suggesting that the initial impairments of the cardiac functions were comparable (Figures 2c and 2d).

### HUVECs-derived exosomes improved cardiac function and myocardial pathological changes in AMI mice

**Figure 3.**
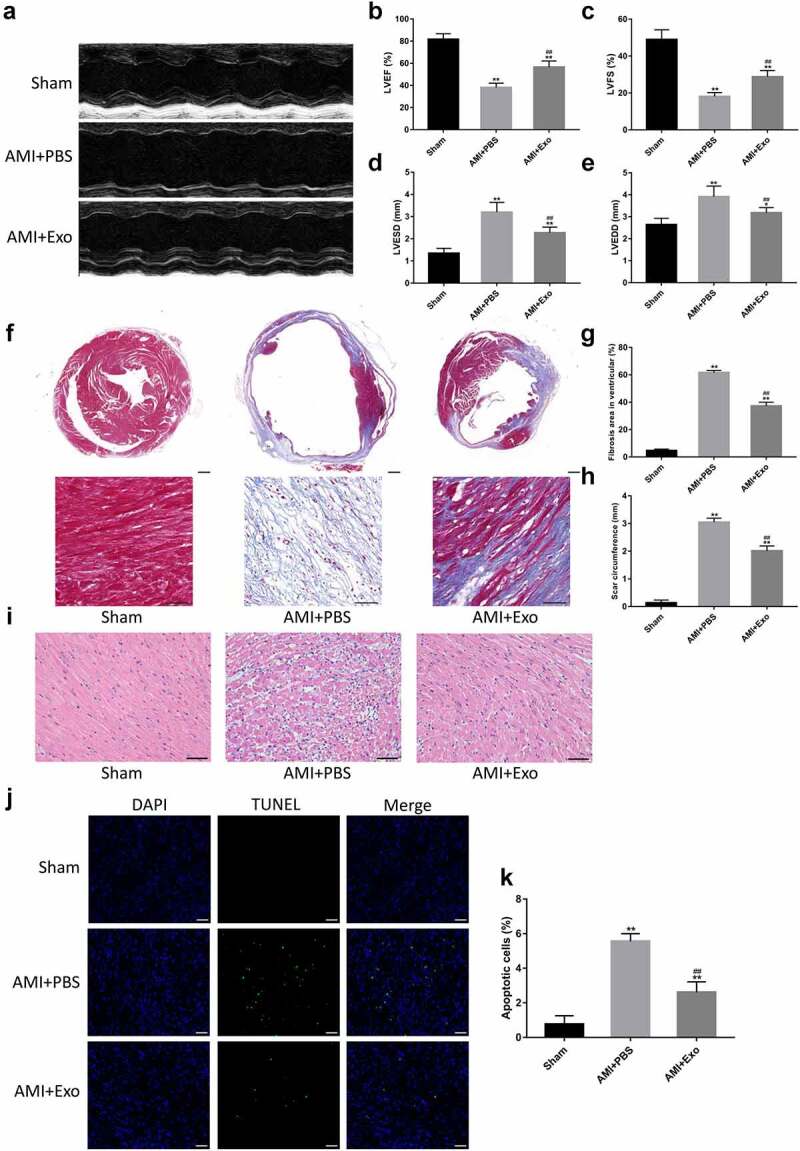
HUVECs-derived exosomes improved cardiac function and myocardial pathological changes in AMI mice. (a) Representative M-mode echocardiographs in short axis using transthoracic echocardiography. (b-e) Cardiac function-related indicators, including LVEF, LVFS, LVESD, and LVEDD (n = 6 per group). (f-h) Quantification of fibrosis area and scar circumference in left ventricular after Masson’s trichrome staining (scale bar = 200 µm or 50 µm) (n = 6 per group). (i) Pathological changes in the cardiac tissues in HE staining (scale bar = 50 µm). (j-k) Cardiomyocyte apoptosis in the mice’s infarcted hearts was tested by TUNEL staining (scale bar = 50 µm) (n = 6 per group). Statistical data were described as mean ± SD, ANOVA was used followed by LSD post hoc least significant difference test; *P < 0.05, **P < 0.01 vs. Sham group; ##P < 0.01 vs. AMI + PBS group. Abbreviations: AMI, acute myocardial infarction; PBS, phosphate-buffered saline; Exo, exosome; LVEF, left ventricular ejection fraction; LVFS, left ventricular fractional shortening; LVESD, left ventricular end-systolic diameter; LVEDD, left ventricular end-diastolic diameter; DAPI, 4’,6-diamidino-2-phenylindole; and TUNEL, terminal deoxynucleotidyl transferase (TdT)-mediated dUTP nick-end labeling.

To confirm whether the delivery of HUVECs-derived exosomes can improve cardiac function after AMI, transthoracic echocardiography was performed after 28 days of surgery. The results demonstrated that the contraction of left ventricular anterior walls in the AMI + PBS group mice was weaker than in the Sham group. On the other hand, a more muscular contraction was observed in the AMI + Exo group than in the AMI + PBS group (Figure 3a). The LVEF, LVFS, LVESD, and LVEDD were further assessed. Briefly, the LVEF and LVFS significantly decreased, while the LVESD and LVEDD increased dramatically in the AMI + PBS group compared to the Sham group. However, these cardiac function-related parameters were improved considerably in the AMI + Exo group compared to the AMI + PBS group, showing an increased LVEF and LVFS and a decreased LVESD and LVEDD (Figures 3b-3e). To further evaluate the therapeutic potential of exosomes on fibrosis after AMI, the fibrosis areas and scar circumference in each group were measured using Masson’s trichrome staining. The data revealed that the fibrosis area and scar circumference increased dramatically in the AMI + PBS group compared to the Sham group. In contrast, those in the AMI + Exo group decreased significantly compared to the AMI + PBS group (Figures 3f-3h). Additionally, the HE staining showed that the cardiomyocytes in the Sham group exhibited a regular arrangement with low infiltration of inflammatory cells in the spaces between cardiomyocytes. In contrast, those in the AMI + PBS group were disarrayed with plenty of necrosis, edema, and infiltration of inflammatory cells. These pathological changes in cardiac tissues were significantly alleviated in the AMI + Exo group (Figure 3i). The cardiomyocyte apoptosis was tested using TUNEL staining, which indicated plentiful apoptotic cells in the AMI + PBS group compared to the Sham group. However, the cardiomyocyte apoptosis was reduced in the AMI + Exo group compared to the AMI + PBS group (Figures 3j and 3k).

*HUVECs-derived exosomes attenuated hypoxia-induced apoptosis and exerted cardioprotective effects in H9C2 cells*

**Figure 4.**
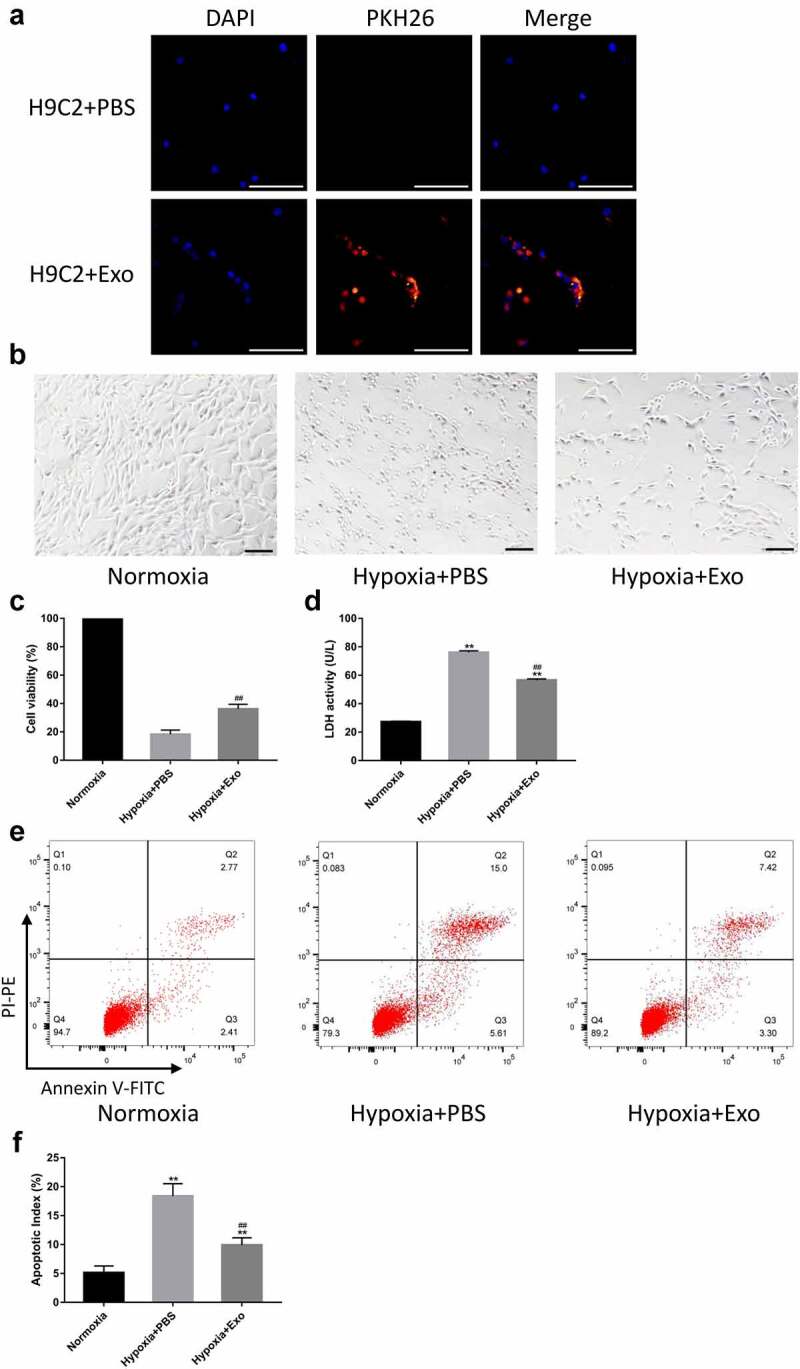
HUVECs-derived exosomes attenuated hypoxia-induced apoptosis and exerted cardioprotective effects in H9C2 cells. (a) HUVECs-derived exosomes labeled with PKH26 internalized by H9C2 cells (scale bar = 500 µm). (b) Morphology of H9C2 cells incubated with HUVECs-derived exosomes or PBS observed under a light microscope (scale bar = 100 µm). (c) H9C2 cells viability was assessed using CCK-8 with 100% viability of cells in a normal environment (n = 6 per group). (d) Lactate dehydrogenase (LDH) release from H9C2 cells was tested using an LDH Cytotoxicity Assay Kit (n = 6 per group). (e-f) Flow cytometry was performed to evaluate the apoptosis of H9C2 cells (n = 6 per group). Statistical data were described as mean ± SD, ANOVA was used followed by LSD post hoc least significant difference test; **P < 0.01 vs. Normoxia group; ##P < 0.01 vs. Hypoxia + PBS group. Abbreviations: DAPI, 4’,6-diamidino-2-phenylindole; PBS, phosphate-buffered saline; Exo, exosome; LDH, lactate dehydrogenase; and FITC, fluorescein isothiocyanate.

To visualize the uptake of exosomes, HUVECs-derived exosomes were labeled using the PKH26 Red Fluorescent Cell Linker Kit and co-cultured with H9C2 cells. The result showed that most of the HUVECs-derived exosomes were internalized by the cells to their cytoplasm, some of which could even enter the nucleus (Figure 4a). The H9C2 cells were incubated with PBS or HUVECs-derived exosomes and placed in a normal or hypoxic environment for 24 h. The morphology of H9C2 cells observed under the light microscope showed that H9C2 cells in the Hypoxia + PBS group were round and shrank compared to the Normoxia group, which were significantly attenuated by HUVECs-derived exosomes (Figure 4b). CCK assay revealed a significant decrease in cell viability in the Hypoxia + PBS group compared to the Normoxia group. In contrast, the reduction in Hypoxia + Exo group was lower than that in the Hypoxia + PBS group (Figure 4c). The cardioprotective effects of HUVECs-derived exosomes were further confirmed using the LDH cytotoxicity assay. The results demonstrated that the release of LDH from H9C2 cells in the Hypoxia + PBS group increased compared to the Normoxia group, while that in the Hypoxia + Exo group was lower than the Hypoxia + PBS group (Figure 4d). To investigate the effects of HUVECs-derived exosomes on the apoptosis of H9C2 cells, flow cytometry was performed using a FITC Annexin V Apoptosis Detection Kit I. The flow cytometry showed that the apoptosis of H9C2 cells in the Hypoxia + PBS group increased compared to the Normoxia group. Nevertheless, HUVECs-derived exosomes could reduce the apoptosis of H9C2 cells under a hypoxic environment (Figures 4e and 4f).

### HUVECs-derived exosomes reduced apoptosis and activated the PI3K/AKT signaling pathway in AMI mice

**Figure 5.**
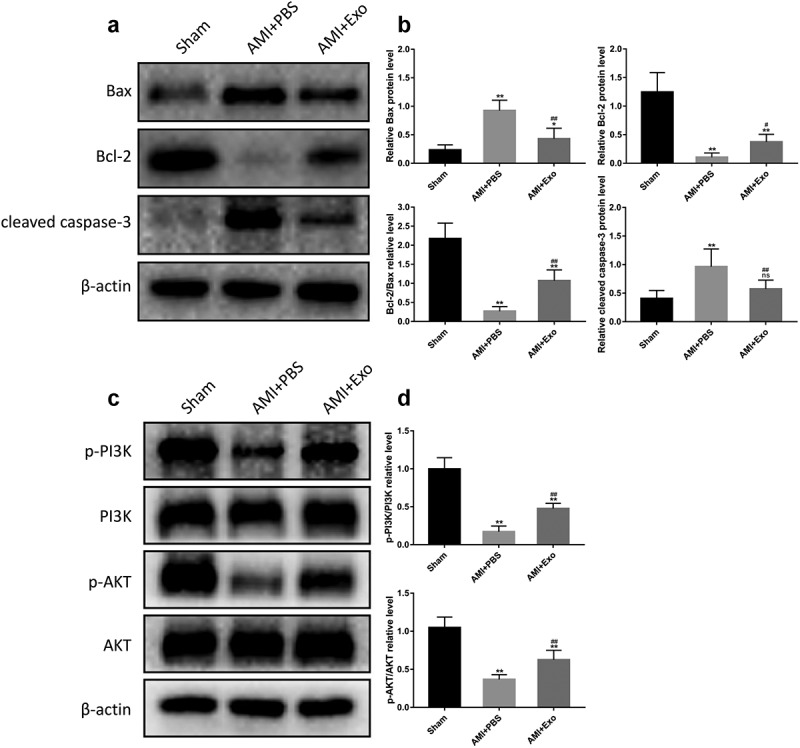
HUVECs-derived exosomes reduced apoptosis and activated the PI3K/AKT signaling pathway in AMI mice. (a) Representative western blot bands of Bax, Bcl-2, and cleaved caspase-3. (b) Quantitative analysis of the levels of Bax, Bcl-2, and cleaved caspase-3 (n = 6 per group). (c) Representative western blot bands of p-PI3K, PI3K, p-AKT, and AKT. (d) Quantitative analysis of p-PI3K/PI3K and p-AKT/AKT (n = 6 per group). Statistical data were described as mean ± SD, ANOVA was used followed by LSD post hoc least significant difference test; *P < 0.05, **P < 0.01 vs. Sham group; #P < 0.05, ##P < 0.01 vs. AMI + PBS group, ns meant no significance vs. Sham group. Abbreviations: AMI, acute myocardial infarction; PBS, phosphate-buffered saline; Exo, exosome; Bax, B Cell Lymphoma-2-associated X protein; Bcl-2, B Cell Lymphoma-2; p-PI3K, phosphorylated phosphatidylinositol 3-kinase; PI3K, phosphatidylinositol 3-kinase; p-AKT phosphorylated protein kinase B; AKT, protein kinase B; and ns, no significance.

To further explore the effects of HUVECs-derived exosomes on apoptosis in AMI mice, the expression levels of apoptosis-related proteins, including Bax, Bcl-2, and cleaved caspase-3, were detected. Western blot analysis indicated that, in the AMI + PBS group, as compared with the Sham group, the expression levels of Bax and cleaved caspase-3 were dramatically up-regulated. At the same time, that of Bcl-2 was significantly down-regulated. Inversely, HUVECs-derived exosomes treatment elevated the Bcl-2 expression level and reduced the Bax and cleaved caspase-3 expression levels (Figures 5a and 5b). Having a close relationship with the PI3K/AKT signaling pathway, apoptosis was further evaluated by the expression levels of crucial signal proteins in this pathway in each group. Western blot analysis showed that the expression levels of p-PI3K and p-AKT in the AMI + PBS group decreased significantly compared to the Sham group. Conversely, HUVECs-derived exosomes treatment effectively promoted the expression levels of p-PI3K and p-AKT (Figures 5 c and 5d).

## Discussion

In this study, we investigated the effects and mechanism of HUVECs-derived exosomes on AMI. The results revealed that the localized injection of HUVECs-derived exosomes in the infarcted area could significantly attenuate fibrosis after AMI, enhance cardiac function and inhibit cardiomyocyte apoptosis, which might be regulated through PI3K/AKT signaling pathway.

Cell therapy is a promising approach for AMI. Various studies have demonstrated that mesenchymal stromal cells, embryonic stem cells, and endothelial progenitor cells were used as a source of precursors in the repair of infarcted myocardium [25]. Previous research has revealed that HUVECs possess a high degree of plasticity and stem cell-like potential. They can differentiate into diverse types of cells, such as cardiomyocytes [26], neuron-like cells [27], and vascular smooth muscle cells [28]. When HUVECs are co-cultured with other cells, they can affect both cell types’ proliferation speeds and differentiation [29,30]. Moreover, the transplantation of HUVECs can restore left ventricular function and ameliorate the pathologic myocardial remodeling after AMI [15,16]. Despite early enthusiasm, cell therapy has obtained modest clinical benefits. However, it has numerous limitations, such as tumorigenicity, immunological rejection, and low cell survival rate after transplantation [31]. Accumulating evidence from both basic and clinical research suggests that cell therapy’s powerful agents are mainly exerted by the paracrine signaling of cells, especially exosomes [32]. Exosomes are double-layered lipid vesicles with a diameter of 30–150 nm, containing various cargo molecules. They can directly contact the target organs and be easily internalized by the target cells. Besides, exosomes have significant advantages, including the same cardioprotective effects as their parental cells, low tumorigenicity, and low immunogenicity [33]. These distinctive characteristic features make exosomes therapy more attractive than cell therapy. A great deal of investigations has illustrated that exosomes released by several types of cells, such as mesenchymal stromal cells and embryonic stem cells, play a significant role in cardiac repair after AMI through inhibiting apoptosis, promoting angiogenesis and cardiomyocyte proliferation [34]. Whereas whether HUVECs-derived exosomes are involved in AMI remains unknown. A study has suggested that HUVECs-derived exosomes can significantly facilitate the formation of neurospheres in primary mouse neural stem cells and reduce apoptosis [35]. The tail vein injection of HUVECs-derived exosomes limits the area of cerebral infarction in mice with middle cerebral artery occlusion and protects neurons from ischemia injury [36]. This evidence provides a basis for HUVECs-derived exosomes to protect the cardiac tissues against ischemia injury. In our study, we found that the localized injection of HUVECs-derived exosomes could reduce the fibrosis area and restore cardiac function after AMI. In addition, HUVECs-derived exosomes alleviated the pathological changes in cardiac tissues, such as myocardial necrosis and inflammatory infiltration.

To identify whether apoptosis is functionally involved in the cardioprotective effects of HUVECs-derived exosomes. We conducted both *in-vivo* and *in-vitro* experiments to evaluate the apoptosis in the AMI mouse models and hypoxic H9C2 cells. Apoptosis, the main form of cell death, refers to programmed cell death under gene regulation, which maintains the homeostasis of the internal environment [37]. Apoptosis significantly conduces to cardiomyocyte cell death and accounts for the increasing loss of cardiomyocytes during the subacute phase of myocardial infarction [4]. It has a relationship with parameters of progressive LV remodeling and the worsening of heart failure. In addition, patients diagnosed with symptomatic heart failure shortly post AMI are connected with obviously increased apoptotic index [38]. Inhibiting apoptosis can effectively control the progression of the disease and improve the poor prognosis of patients with AMI [39]. In our study, we found the inhibitory effects of HUVECs-derived exosomes on apoptosis. They could protect the myocardium from apoptosis induced by AMI in mice and reduce the apoptosis of H9C2 cells in a hypoxic environment. Besides, apoptosis-related proteins were detected in this investigation. Bax, Bcl-2, and caspase-3 are vital regulators involved in apoptosis progression. When cells receive the stimulation of a dead signal, Bax is translocated to the mitochondrial membrane and forms homodimers or multimers themselves. Then, the mitochondrial permeability transition pore (mPTP) is facilitated opening, causing a release of cytochrome *c* [40]. Caspase 3 is the most critical executioner downstream caspase cascade. Its activation relies heavily on the release of cytochrome *c*. Once activated, intracellular proteins are degraded massively, leading to irreversible cell death [41]. Bcl-2 can interact with Bax to form heterodimers, preventing mitochondrial permeabilization and apoptosis [42]. We observed that the expression levels of Bax and cleaved caspase-3 were increased, whereas the Bcl-2 expression level was reduced after AMI. Reversely, HUVECs-derived exosomes decreased the Bax and cleaved caspase-3 levels and elevated the Bcl-2 level. The results revealed that HUVECs-derived exosomes significantly restored the AMI-induced disorders of these apoptosis-related proteins. These findings demonstrated that HUVECs-derived exosomes might exert their cardioprotective effects via inhibiting apoptosis.

Interestingly, PI3K/AKT signaling pathway has been reported to participate in cell growth, differentiation, proliferation, and survival [43]. PI3K consists of catalytic subunit p110 and regulatory subunit p85. Extracellular signals can make the SH2 domain of subunit p85 of PI3K phosphorylated. AKT is a vital downstream protein of the PI3K signaling pathway, and it plays an important role in anti-apoptosis [44]. Phosphorylated PI3K phosphorylates PIP2 (phosphatidylinositol 4,5-bisphosphate) to PIP3 (phosphatidylinositol 3,4,5-trisphosphate). Then, PIP3 is combined with the PH domain of AKT, and this enables 3-phosphatidylinositol-dependent protein kinase (PDK1) to phosphorylate AKT further. Activated AKT increases the level of Bcl-2 to inhibit the oligomerization of Bax or keep the caspase inactivated, thereby exerting anti-apoptotic effects [45]. Our results found that HUVECs-derived exosomes increased the expression levels of p-PI3K, p-AKT, and Bcl-2 and decreased the expression levels of Bax and cleaved caspase-3 in AMI mice. This suggested that apoptosis was negatively associated with PI3K/AKT signaling in AMI, demonstrating the therapeutic effects of HUVECs-derived exosomes. Another downstream target of the PI3K/AKT signaling pathway is glycogen synthase kinase 3 (GSK-3). GSK-3 family possesses two subtypes, including GSK-3α and GSK-3β. They were both expressed in the heart [46]. Sufficient evidence has demonstrated that GSK-3 is regulated by PI3K and AKT. Phosphorylated AKT can phosphorylate GSK-3 and inactive them. Besides, overexpression of active GSK-3 facilitates apoptotic cell death, whereas inhibiting the expression of active GSK-3 prevents apoptosis [47]. Whether GSK-3α/β is involved in the cardiac protective effects of HUVECs-derived exosomes on AMI needs to be further discussed in the future. There were several limitations in this study. First, this study has not fully elucidated the effects of HUVECs-derived exosomes on improving myocardial injury through other signaling pathways. Second, the inhibitors for PI3K were not utilized in this study to confirm that HUVECs-derived exosomes indeed exerted myocardial protection by activating the PI3K/AKT signaling pathway. We will try our best to solve these problems in the future. The exosomes therapy was used based on cell therapy, which effectively avoided the problems caused by cellular transplantation. This study might provide a novel idea for the treatment of AMI.

## Conclusions

In summary, this study indicated that HUVECs-derived exosomes could effectively enhance cardiac function after AMI and inhibit cardiomyocyte apoptosis, which might be regulated through the PI3K/AKT signaling pathway. This study might provide a theoretical basis for HUVECS-derived exosomes as a novel treatment strategy for AMI.

## Supplementary Material

Supplemental Material

## Highlights

• HUVECs-derived exosomes have cardioprotective effects against AMI.
• HUVECs-derived exosomes inhibited cardiomyocyte apoptosis during AMI in mice.
• PI3K/AKT signaling pathway was activated in AMI mouse models treated with HUVECs-derived exosomes.
