# Supplementary figures and images for: Human umbilical vein endothelial cells-derived exosomes enhance cardiac function after acute myocardial infarction by activating the PI3K/AKT signaling pathway
Source: Bioengineered. 2022 Mar 31;13(4):8850–65. doi: 10.1080/21655979.2022.2056317 (PMC9161948; doi:10.1080/21655979.2022.2056317)

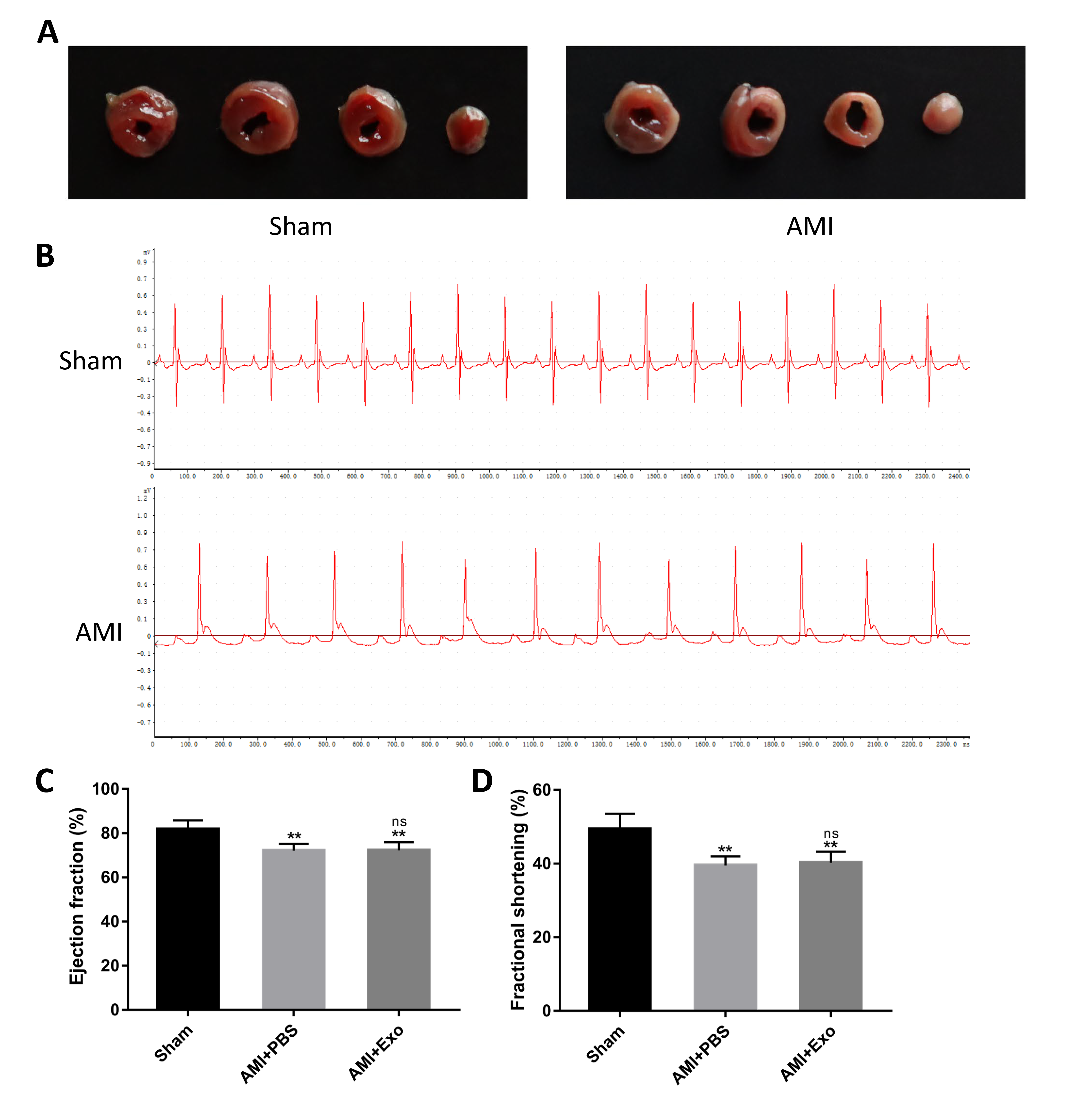

Supplement: Supplemental Material [file KBIE_A_2056317_SM9659.zip › Figure2.tif]
